# Need for psychiatric phenotyping in patients with rare genetic disorders

**DOI:** 10.1007/s00787-021-01761-2

**Published:** 2021-03-27

**Authors:** Franziska Degenhardt, Gertraud Gradl-Dietsch, Johannes Hebebrand

**Affiliations:** Department of Child and Adolescent Psychiatry, Psychosomatics and Psychotherapy, University Hospital Essen, University of Duisburg-Essen, Essen, Germany

Genetic information is increasingly part of child and adolescent psychiatry. The number of publications in *European Child and Adolescent Psychiatry* on genetic topics reflects this*.* For example, recent papers focused on patients with a genetic diagnosis (e.g. 22q11.2 deletion syndrome) [1, 2] and parents of a child with a genetic disorder [3]. Furthermore, clinicians routinely interact with patients with familial high risk for a mental health disorder [4]. A recent *European Child and Adolescent Psychiatry* editorial pinpointed the integration of genetics into this medical specialty as a major topic within future training of child and adolescent psychiatrists [5, 6].

In the past, genetic testing to confirm or rule out a specific genetic diagnosis was rarely applied in routine clinical child and adolescent psychiatry. If at all, limited genetic testing was offered to patients with intellectual disability (ID) and/or clinical symptoms indicative of a specific underlying genetic syndrome. Examples for the latter include Trisomy 21 (Down syndrome), Rett-syndrome, and fragile (X)-syndrome. It was well understood that these syndromes represent the *tip of the iceberg.* Thus, the expectation was that many more genetic factors underlying ID or mental disorders with and without additional specific clinical symptoms awaited identification, once the technology for a systematic analysis of the genome was available.

Over the past ten years, large-scale international science collaborations and major technological advances in the characterization of the human genome facilitated the detailed analysis of the genetic architecture of psychiatric disorders [7]. Currently, the largest body of genetic data exists for ID and autism spectrum disorder (ASD). Now, it is well established that ID and ASD—in addition to their clinical heterogeneity—are genetically heterogeneous. ID and ASD have a complex genetic architecture. They are brain disorders with both common and rare variants in more than 1000 genes contributing to their development [8, 9]. In a small subgroup of patients rare monogenic disorders, chromosomal abnormalities and specific copy number variants may underlie these phenotypes, the overarching similarity being the very strong effect size of the underlying genetic aberration. This is the patient subgroup that will currently benefit from genetic testing in the clinical context. For a large proportion of patients with ID and ASD (comparable with other mental disorders), the combination of common and infrequent variants in multiple genes will be the largest genetic factor contributing to disease etiology. Every one of these disorders is characterized by the additive (and potentially non-additive) effects of specific variants at more than a thousand loci, with each variant having only a small effect size [8]. In combination with environmental factors (and potentially other genetic factors yet to be identified), the combined effect of these variants results in the respective phenotype. Currently, analyzing common variants in patients with ID/ASD is clinically not meaningful, because the explained variance is too small. However, this is a completely different story for rare pathogenic variants.

The latter include rare copy number variants (CNVs; deletions or duplications of genetic material compared to the reference genome) and single base pair changes detected using next-generation sequencing (exome or whole genome sequencing). Genome-wide CNV screening (chromosomal microarray analysis, CMA) and/or exome sequencing (sequencing of the coding region of all genes) are now recommended as part of the diagnostic work-up of patients with ID and/or ASD [9–11]. On average, the diagnostic yield of CMA is 10% in individuals with ASD and 25% in individuals with ID. Combining CMA with exome sequencing increases the diagnostic yield to approximately 30–40% [10–12].

Patients and their parents/caregivers may benefit from receiving a molecular genetic diagnosis in several ways; (i) The *diagnostic odyssey* in referrals to potentially multiple clinical specialities trying to make sense of a broad range of symptoms in multiple organs comes to an end [13; genetic syndromes generally affect more than one organ]. (ii) Patients and parents obtain access to syndrome-specific support groups tailored to their needs and interests. (iii) The genetic diagnosis may entail more informed somatic and mental health care. For instance, patients with a diagnosed 22q11.2 deletion syndrome should be evaluated by a cardiologist (for assessment and treatment of congenital heart disease [14]. The genetically increased risk for impaired kidney function in patients with a 17q12 deletion should inform the physician to avoid nephrotoxic medications [15]. (iv) Finally, particularly parents, who have brooded as to why their child shows specific mental symptoms, may find relief in discovering that a genetic disorder underlies these symptoms.

As child and adolescent psychiatrists, we will be most interested in how a genetic diagnosis underlying ID/ASD might change the psychiatric care of our patients. Particularly for rare genetic disorders, the respective gain in knowledge is currently very limited and oftentimes anecdotal. There are several reasons for this: (i) the discovery of rare, clinically relevant variants with intermediate to strong effect sizes requires very large sample sizes [7, 8]. The impressively large cohorts are gathered through international collaborations and these oftentimes do not publish detailed phenotypic information on e.g. specific CNV carriers; (ii) in the clinical context, genetic testing in children with ID/ASD is mainly ordered by non-psychiatrists (e.g. paediatricians, clinical geneticists). Therefore, the majority of published case reports/series pertaining to specific genetic diagnoses do not include detailed information on the patients’ mental status and history; (iii) Furthermore, psychiatric phenotyping using different behavioural assessment batteries requires expertise and is time-consuming.

The leading example (and currently one of only a few) on how a genetic diagnosis informs psychiatric care, are deletions in the chromosomal region 22q11.2. For more than 30 years, CNVs in this chromosomal region have been the focus of scientific and clinical studies. Deletions in 22q11.2 are a known cause for both ID and ASD [14]. Furthermore, CNV carriers have a substantially increased risk for several other mental disorders (including schizophrenia, attention-deficit/hyperactivity disorder, mood disorders, and anxiety disorders; 14). Patients with a 22q11.2 deletion and psychosis respond well to clozapine [16]. However, both clozapine and the deletion decrease the seizure threshold and therefore taking this atypical antipsychotic drug in combination with an antiepileptic drug should be considered [17].

As child and adolescent psychiatrists, we will increasingly interact with families of a child diagnosed with a specific genetic disorder and accordingly will be faced with questions in relation to syndrome-specific psychiatric management. It is invaluable to collaborate with self-help groups to come up with appropriate treatments for specific symptoms as for example insomnia (frequent symptom in patients with a genetic disorder). Such treatment schemes may provide guidance even if the empirical evidence is scant. To improve our patient’s care, collecting and sharing information on the psychiatric phenotype and treatment of patients diagnosed with a genetic syndrome is essential.

A further aspect requires delineation. Every identified gene/genes relevant to mental disorder sheds light into the disorder’s etiology. Thus, a genetic variant with a strong effect size may entail the elucidation of one or more pathways involved in patients with the same psychiatric diagnosis but without the specific genetic diagnosis. A cross-check of findings obtained in genome-wide association studies within the genetic region of interest may lead to the discovery that common variants (single nucleotide polymorphisms) in the locus of interest contribute to the polygenic architecture of the respective disorder. In conclusion, each genetic finding offers the opportunity to advance our knowledge of how genetic factors can entail the respective phenotype.

*European Child and Adolescent Psychiatry* aims at addressing this need by establishing a specific section for case reports/series of patients with a mental disorder diagnosed with a genetic disorder. The published case reports/series will focus on the mental phenotype of patients with a rare genetic disorder. It needs to include solid data on the mental phenotype based on at least one unpublished patient in combination with a brief review (if available) of all published patients (for details see Genetic case reports/series in instructions for authors (website). We hope that the times are over during which patients were plainly characterized as being *hyperactive* or as having *behavioural abnormalities*. We owe it to our patients, their families, and to ourselves to advance this important field by improving the phenotyping of such genetic disorders. There is much to learn. As physicians, we need to incorporate such individualized diagnoses and treatments into our clinical routine.
